# Association of serum uric acid-to-high-density lipoprotein cholesterol ratio with cardiovascular disease risk in patients with metabolic dysfunction-associated fatty liver disease: a cross-sectional NHANES analysis

**DOI:** 10.3389/fnut.2025.1561594

**Published:** 2025-04-09

**Authors:** Xianyao Wang, Yuchen Shi, Ying Zi, Jun Long, Rongjie Shi

**Affiliations:** ^1^Department of Gastroenterology, The First Affiliated Hospital of Dali University, Dali, Yunnan, China; ^2^The First Affiliated Hospital of Kunming Medical University, Kunming, Yunnan, China

**Keywords:** serum uric acid-to-high-density lipoprotein cholesterol ratio (UHR), metabolic dysfunction-associated fatty liver disease (MAFLD), cardiovascular risk, Framingham Risk Score (FRS), NHANES

## Abstract

**Objective:**

The relationship between the serum uric acid-to-high-density lipoprotein cholesterol ratio (UHR) and cardiovascular disease (CVD) risk in patients with metabolic dysfunction-associated fatty liver disease (MAFLD) is unknown. This study aims to investigate the relationship between UHR and cardiovascular disease risk in patients with MAFLD.

**Methods:**

Data for this study were obtained from the National Health and Nutrition Examination Survey (NHANES) 2017–2020, in which 3289 patients with MAFLD participated. Participants were grouped according to their 10-year cardiovascular disease risk level, which was assessed by the Framingham Risk Score (FRS). We used binary logistic regression to analyze the relationship between UHR and CVD risk and smoothed curve-fitting models and threshold effect analyses to describe the relationship between UHR and CVD risk scores.

**Results:**

After adjusting for all confounders, individuals with high UHR exhibited a higher prevalence of intermediate/high risk by FRS [odds ratio (OR): 2.12, 95% confidence interval (CI): (1.34, 3.35), *P* = 0.001]. UHR was nonlinear positive correlated with FRS (log-likelihood ratio test < 0.001). And there was a breakpoint of 364.38 and an apparent threshold effect. When UHR was lower than 364.38, The FRS increased with increasing UHR (*P* < 0.0001), whereas when UHR was higher than 364.38, the relationship between FRS and UHR was statistically insignificant (*P* = 0.0964).

**Conclusion:**

The UHR was significantly associated with a 10-year risk of cardiovascular disease in patients with MAFLD. Higher UHR was associated with higher FRS in patients with MAFLD. The UHR can be a valid biomarker for predicting the 10-year risk of cardiovascular disease in patients with MAFLD.

## Introduction

As noted by an international expert consensus conference, metabolic dysfunction-associated fatty liver disease (MAFLD) is a form of hepatic steatosis accompanied by overweight/obesity, type 2 diabetes mellitus, or metabolic dysfunction (1). MAFLD, formerly known as non-alcoholic fatty liver disease (NAFLD), is the leading cause of chronic liver disease worldwide, and its prevalence has been increasing, posing a significant health and economic burden on society (2). By far, cardiovascular disease is the leading cause of death worldwide (3). A meta-analysis confirmed that the incidence of CVD was 2.26 times higher in the MAFLD group than in the non-MAFLD group, and the risk of CVD death was significantly higher in the MAFLD group (4). Currently, cardiovascular disease remains the leading cause of death in the MAFLD population, even more than liver disease itself, posing a serious threat to human health and a significant healthcare burden (5). Therefore, early assessment of the future risk of cardiovascular disease in patients with MAFLD and early intervention can help to improve the quality of life and reduce the risk of death in patients with MAFLD.

The serum uric acid-to-high-density lipoprotein cholesterol ratio (UHR) is a recently proposed marker of inflammation and metabolism that utilizes serum uric acid (SUA) and high-density lipoprotein cholesterol (HDL-C) to provide a cost-effective and readily available indicator that better responds to disturbances in the body’s glucose-lipid metabolism. The UHR has been shown to be associated with the risk of developing fatty liver (6, 7), diabetes (8), diabetic kidney injury (9), insulin resistance (10, 11) and metabolic syndrome (12). The Framingham Risk Score (FRS) is a tool to assess the risk of cardiovascular disease over the next decade based on certain risk factors (13). However, to date, no study has investigated the relationship between UHR and CVD risk in patients with MAFLD. Therefore, this study aimed to investigate the correlation between UHR and 10-year CVD risk (assessed based on FRS) in patients with MAFLD, thus providing a valuable reference for the prevention and management of CVD in patients with MAFLD.

## Materials and methods

### Study population

**FIGURE 1:**
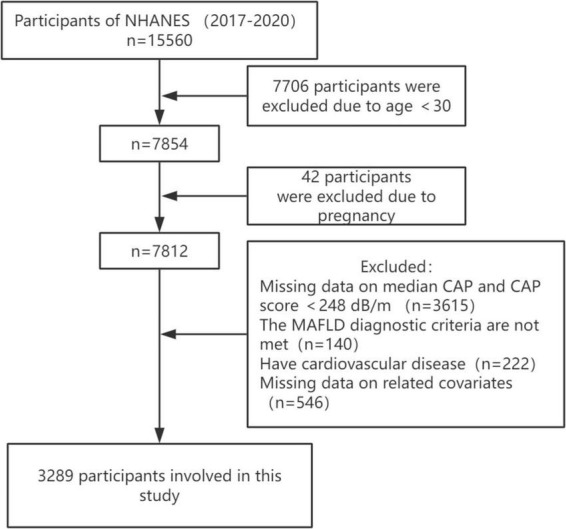
Flow chart of the study.

Our study data come from the National Health and Nutrition Examination Survey (NHANES), the most in-depth survey administered by the National Center for Health Statistics (NCHS) to assess the health and nutritional status of adults and children in the United States. NHANES surveys approximately 5,000 people in 15 different counties across the country each year on a 2-year cycle, and through sample-weighted analyses, the study cohort is representative of the entire United States population. All participants provided written informed consent. This study is based on data from NHANES from March 2017 to 2020, a cycle that includes participants’ vibration-controlled transient elastography (VCTE) data used to define MAFLD. Based on a large meta-analysis with a controlled attenuation parameter (CAP) of ≥248 dB/m (AUC: 0.823, Sensitivity: 0.688, Specificity: 0.822) was used as the cut-off value for the diagnosis of hepatic steatosis (14). A median liver stiffness of ≥8.2 kPa was considered significant fibrosis (15). Out of 15,560 participants, those who were less than 30 years old, pregnant, those with missing CAP data and CAP less than 248 dB/m, those who did not meet the diagnosis of MAFLD, those with missing data on relevant covariates, and those who suffered from cardiovascular diseases (congestive heart failure, coronary artery disease, angina pectoris, heart attack, and stroke) were excluded, and finally, a total of 3,289 participants were enrolled in the study (Figure 1).

### Definition of MAFLD

Based on the presence of imaging evidence of hepatic steatosis in combination with one of the following three conditions: overweight or obesity (defined as BMI ≥ 25 kg/m2 for Caucasians and BMI ≥ 23 kg/m2 for Asians), type 2 diabetes mellitus, and metabolic dysfunction. Metabolic dysfunction was defined as the presence of at least two of the following risk factors for metabolic abnormalities: (1) waist circumference ≥ 102 cm in Caucasian males and ≥88 cm in females (or ≥90 cm in Asian males and ≥80 cm in females); (2) blood pressure ≥ 130/85 mmHg or antihypertensive medication; (3) triglyceride (TG) ≥ 1.7 mmol/L or lipid-lowering medication; (4) high-density lipoprotein cholesterol (HDL-C) < 1.0 mmol/L in men and HDL-C < 1.3 mmol/L in women; (5) pre-diabetes; (6) insulin resistance index (HOMA-IR) ≥ 2.5; and (7) ultrasensitive C-reactive protein (hs-CRP) > 2 mg/L (1).

### Cardiovascular disease risk

The Framingham Risk Score (FRS) was used to assess participants’ cardiovascular disease risk over the next 10 years. The FRS assesses an individual’s 10-year risk of cardiovascular disease based on gender, age, diabetes status, smoking status, treated versus untreated systolic blood pressure, total cholesterol (TC), and HDL-C. The FRS is categorized into three risk classes: low risk (<10%), intermediate risk (10–20%), and high risk (>20%) (13). In this study, we categorized participants as low risk and intermediate/high risk.

### Definition of research variables

UHR = serum uric acid (umol/L)/high-density lipoprotein cholesterol (mmol/L).

### Relevant variable

Variables included in this study were gender, age, race, smoking, body mass index (BMI), diabetes, total cholesterol (TC), triglycerides (TG), high-density lipoprotein cholesterol (HDL-C), serum uric acid (SUA) and Estimated glomerular filtration rate (eGFR). Race was categorized as Mexican American, other Hispanic, non-Hispanic White, non-Hispanic Black, non-Hispanic Asian, and other races. BMI was defined as weight (kg)/height (m) squared, and smoking was defined as more than 100 cigarettes smoked in a lifetime, which was obtained from a questionnaire. The methods of testing for TC, TG, HDL-C, and SUA are described in detail on the official NHANES website. Diabetes mellitus was defined as “your doctor has told you that you have diabetes mellitus,” or fasting blood glucose ≥ 7.0 mmol/L or random blood glucose ≥ 11.1 mmol/L or HbA1c > 6.5%, or taking glucose-lowering medication to reduce blood glucose or using insulin. We use the well-recognized Chronic Kidney Disease Epidemiology Collaboration (CKD-EPI) formula to evaluate eGFR (16).

### Statistical analysis

In our study, all statistical analyses were weighted based on officially recommended weights. Continuous variables were expressed as mean ± standard deviation and categorical variables were expressed using frequency counts and weighted percentages. Weighted linear regression models (for continuous variables), as well as weighted chi-square tests (for categorical variables), were utilized to compare the differences between the two groups. Binary logistic regression was used to analyze the relationship between UHR and CVD risk. Model 1 was unadjusted for variables; model 2 was adjusted for gender, age, and race; and model 3 was adjusted for gender, age, race, BMI, smoking, TC, TG, diabetes, significant fibrosis and eGFR. In addition, stratified analyses were performed according to gender, age, and BMI. Smoothed curve fitting was used to assess the linear relationship between UHR and FRS. Data were analyzed using the R package, EmpowerStats, and Stata, and *P* < 0.05 was considered statistically significant.

## Results

### Baseline characteristics of participants

In this study, 3289 MAFLD participants were enrolled with a mean age of 54.19 years. Based on the 10-year CVD risk level, participants were categorized into a low risk group (*n* = 1389) and an intermediate/high risk group (*n* = 1900), and the general characteristics of the participants are described in Table 1. The mean age of the participants was significantly different from the mean age of the participants. There was a statistically significant difference in mean age between the two groups (low risk group: 45.54 ± 11.01 vs. intermediate/high risk group: 62.02 ± 10.44, *p* < 0.001). In addition, participants in the intermediate/high risk group were more likely to be male (low risk group: 42.03% vs. intermediate/high risk group: 63.19%, *P* < 0.001), had a higher percentage of smoking (low risk group: 31.26% vs. intermediate/high risk group: 58.19%, *P* < 0.001), and had a higher TG (low risk group: 1.70 ± 1.01 vs. intermediate/high risk group: 2.09 ± 1.60, *P* < 0.001), SUA (low-risk group: 329.09 ± 83.41 vs. intermediate/high risk group: 346.40 ± 83.55, *P* < 0.001), UHR (low risk group: 268.36 ± 106.49 vs. intermediate/high risk group: 299.88 ± 130.78, *P* < 0.001), lower HDL-C (low risk group: 1.33 ± 0.36 vs. intermediate/high risk group: 1.26 ± 0.37, *P* < 0.001), eFFR (low risk group: 95.32 ± 17.27 vs. intermediate/high risk group: 82.04 ± 18.85, *P* < 0.001), higher prevalence of diabetes mellitus (low risk group: 9.15% vs. intermediate/high risk group 36.72%, *P* < 0.001), and significant prevalence of significant fibrosis (low risk group: 12.44% vs. intermediate/high risk group 17.74%, *P* < 0.001). And there was no statistical difference in TC between the two groups (*P* = 0.311).

**TABLE 1:** The baseline characteristics of participants (weighted).

Variable	Total (*n* = 3289)	Low risk (*n* = 1389)	Intermediate/high risk (*n* = 1900)	*P*-value
Age(years)	54.19 ± 13.51	45.54 ± 11.01	62.02 ± 10.44	<0.001
Gender,*n*(%)				<0.001
Male	1705(53.13)	512(42.03)	1193(63.19)	
Female	1584(46.87)	877(57.97)	707(36.81)	
Race/ethnicity,*n*(%)				0.001
Mexican American	437(8.71)	213(10.65)	224(6.96)	
Other Hispanic	368(7.26)	149(7.98)	219(6.92)	
Non-Hispanic White	1194(65.47)	464(61.97)	730(68.63)	
Non-Hispanic Black	776(8.97)	326(9.45)	450(8.53)	
Non-Hispanic Asian	363(5.13)	171(5.47)	192(4.82)	
Other race	151(4.46)	66(4.49)	85(4.43)	
Smoking,*n*(%)				<0.001
Yes	1442(45.39)	394(31.26)	1048(58.19)	
No	1847(54.61)	995(68.74)	852(41.81)	
BMI,(Kg/m^2^)	32.90 ± 6.94	33.80 ± 7.59	32.08 ± 6.17	<0.001
TC(mmol/L)	5.00 ± 1.06	4.98 ± 0.94	5.02 ± 1.17	0.311
TG(mmol/L)	1.91 ± 1.37	1.70 ± 1.01	2.09 ± 1.60	<0.001
HDL-C(mmol/L)	1.29 ± 0.37	1.33 ± 0.36	1.26 ± 0.37	<0.001
SUA (umol/L)	338.18 ± 83.93	329.09 ± 83.41	346.40 ± 83.55	<0.001
Diabetes,*n*(%)				<0.001
Yes	975(23.62)	167(9.15)	808(36.72)	
No	2314(76.38)	1222(90.85)	1092(63.28)	
Significant fibrosis,*n*(%)				<0.001
Yes	533(15.22)	172(12.44)	361(17.74)	
No	2756(84.78)	1217(87.56)	1539(82.26)	
eGFR	88.35 ± 19.29	95.32 ± 17.27	82.04 ± 18.85	<0.001
UHR (umol/mmol)	284.90 ± 120.89	268.36 ± 106.49	299.88 ± 130.78	<0.001

Mean ± standard deviation was used to describe continuous variables, and unweighted frequencies and weighted percentages were used to describe categorical variables. *P*-values were calculated using weighted linear regression models for continuous variables and weighted chi-square tests for categorical variables.

### Relationship between UHR and cardiovascular disease risk

**FIGURE 2:**
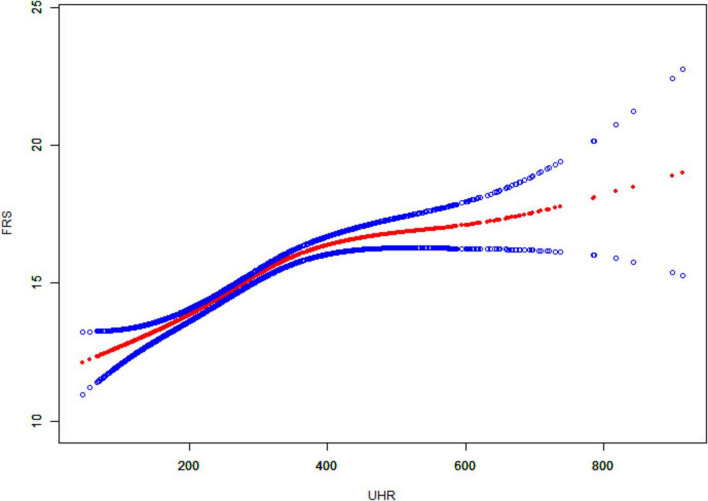
Smooth curve fitting of UHR to FRS. The solid red line curve between the variables. The blue solid line represents the 95% confidence interval of the fitted results.

The association between UHR and CVD risk in MAFLD was analyzed using binary logistic regression models (Table 2). Participants were categorized into low and high UHR groups based on median UHR. In the logistic regression model, individuals with high UHR showed an 85% increase [odds ratio (OR): 1.85, 95% confidence interval (CI): (1.50, 2.29), *P* < 0.001] in the prevalence of intermediate/high risk by FRS, compared to those with low UHR. After adjusting for gender, age, and race, individuals with high UHR still exhibited a higher prevalence of intermediate/high risk by FRS [Model 2: OR. 2.42, 95% CI: (1.71, 3.41), *P* < 0.001]. This relationship did not change after adjusting for gender, age, race, smoking, BMI, TC, TG, diabetes, significant fibrosis and eGFR [Model 3: OR: 2.12, 95% CI: (1.34, 3.35), *P* = 0.001]. In addition, we used a smoothed curve-fitting model and threshold effects analysis to characterize the relationship between UHR and FRS. The results showed that UHR was nonlinearly and positively correlated with FRS (log-likelihood ratio test < 0.001) (Figure 2 and Table 3). There was a breakpoint of 364.38 and an apparent threshold effect. When UHR was lower than 364.38, The FRS increased with increasing UHR (*P* < 0.0001), whereas when UHR was higher than 364.38, the relationship between FRS and UHR was statistically insignificant (*P* = 0.0964) (Table 3).

**TABLE 2:** The association between UHR and cardiovascular risk (weighted).

Variable	Model 1	Model 2	Model 3
	**OR(95%CI) *P*-value**	**OR(95%CI**) ***P*-value**	**OR(95%CI) *P*-value**
**UHR**			
Low UHR	1.00(reference)	1.00(reference)	1.00(reference)
High UHR	1.85 (1.50,2.29) <0.001	2.42 (1.71,3.41) <0.001	2.12 (1.34,3.35) 0.001

Model 1: unadjusted variables. Model 2: adjusted for gender, age, and race. Model 3: adjusted for gender, age, race, BMI, smoking, TC, TG, diabetes, significant fibrosis and eGFR.

**TABLE 3:** Threshold effect.

Outcome	FRS
**Model I**	
A straight-line effect	0.01 (0.01, 0.01) < 0.0001
**Model II**	
Fold points (K)	364.38
<K-segment effect 1	0.01 (0.01, 0.02) < 0.0001
>K-segment effect 2	0.00 (−0.00, 0.01) 0.0964
Effect size difference of 2 vs 1	−0.01 (−0.02, −0.01) 0.0001
Equation predicted values at break points	16.11 (15.48, 16.74)
Log likelihood ratio tests	<0.001

Result variable: FRS. Exposure variable: UHR. Results are expressed as β(95%CI). Adjusted for gender, age, race, smoking, BMI, TC, TG, diabetes, significant fibrosis and eGFR.

### Subgroup analysis

**FIGURE 3:**
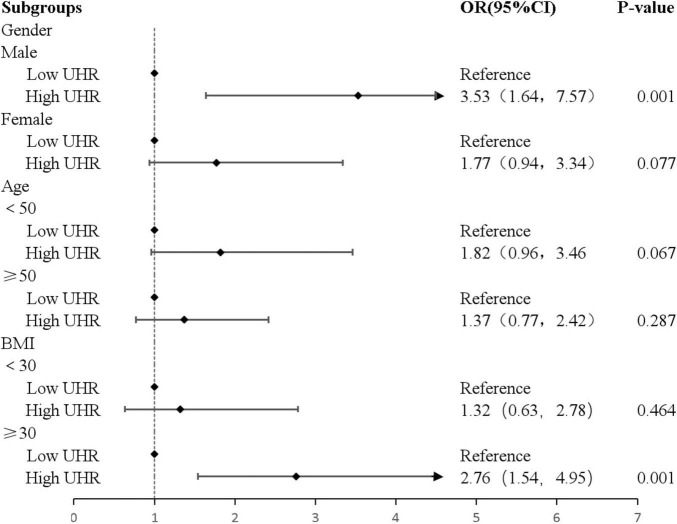
Forest plot of stratified analysis of the correlation between UHR and cardiovascular risk in patients with MAFLD.

We stratified the analysis according to the participants’ gender, age, and BMI. Results showed that in the subgroups of men [OR: 3.53, 95% CI: (1.64, 7.57), *P* = 0.001], and BMI greater than or equal to 30 [OR: 2.76, 95% CI: (1.54, 4.95), *P* = 0.001] subgroups, individuals with high UHR exhibited a higher prevalence of intermediate/high risk by FRS (Figure 3).

## Discussion

A total of 3289 MAFLD patients were enrolled in our study, which showed that high UHR was associated with an increased 10-year risk of cardiovascular disease. Threshold effect model analysis suggested that FRS increased with increasing UHR when UHR was lower than 364.38, while the relationship between FRS and UHR was statistically insignificant when UHR was higher than 364.38. After stratifying by gender, age, and BMI, we found that individuals with high UHR exhibited a higher prevalence of intermediate/high risk by FRS in males, and subjects with a BMI greater than or equal to 30. These findings suggest that UHR may be a valid indicator for assessing 10-year cardiovascular disease risk in patients with MAFLD. To our knowledge, this is the first study to characterize the relationship between UHR and 10-year CVD risk in patients with MAFLD. And the nationally representative data of NHANES was used to enhance the universality.

MAFLD and cardiovascular disease are both common chronic diseases worldwide, imposing a significant health and economic burden on society. There is an association between MAFLD and an increased risk of developing cardiovascular disease, but the mechanisms leading to this increased risk remain unclear. It may be related to elevated levels of inflammation, oxidative stress, and hepatic metabolites (17). The liver is a major regulator of triglyceride metabolism and is responsible for the formation of lipoproteins, including low-density lipoproteins, very low-density lipoproteins, and high-density lipoproteins, to transport cholesterol. Alterations in the production or removal of lipoproteins by the liver can increase the risk of cardiovascular disease (18). Serum uric acid is the metabolic end product of purine nucleotides in the body, produced mainly by the liver and intestine and excreted via the kidneys and digestive tract. Serum uric acid is an important causative risk factor for many cardiovascular diseases. Studies have shown that the risk of cardiovascular disease increases with higher serum uric acid levels and is associated with higher cardiovascular mortality (19, 20). In patients with fatty liver, higher SUA levels were significantly associated with a 10-year risk of cardiovascular disease (21), while higher SUA levels were associated with a higher risk of cardiovascular mortality in patients with fatty liver (22). High serum uric acid increases the risk of cardiovascular disease and may be associated with inflammation, oxidative stress, endothelial dysfunction, and insulin resistance (23).

UHR combines serum uric acid and HDL cholesterol and is an easily measured marker of inflammation and metabolism. A study based on the NHANES database to explore UHR and NAFLD found that elevated UHR levels were independently associated with increased risk of NAFLD and severity of hepatic steatosis (24). In addition, a study from China found a linear positive correlation between UHR and NAFLD risk and that UHR could be used as a non-invasive marker to identify NAFLD risk (25). However, there is still a lack of research on the relationship between UHR and cardiovascular disease risk in patients with fatty liver disease. We associated UHR with CVD risk in patients with MAFLD and found that MAFLD patients with high UHR had a higher 10-year CVD risk. A smooth curve fitting model visualized the correlation between UHR and FRS. The solid red line represents a smoothed curve that illustrates the trend between UHR and FRS. The blue solid line indicates the 95% confidence interval for the fit results, which shows the range of statistical uncertainties associated with the fitted curve. A narrower confidence interval indicates a higher reliability of the fitting results. As can be seen from the figure, FRS increases with the increase of UHR, and a threshold effect model analysis revealed a significant fold point of 364.38. In addition, in subgroup analyses, we found that the association between UHR and CVD risk was more significant in men, and patients with a BMI greater than or equal to 30. The underlying mechanism may be related to sex hormone levels, where higher testosterone levels may inhibit uric acid metabolism, resulting in elevated blood uric acid levels. In addition, obese individuals are often associated with insulin resistance, leading to reduced renal excretion of uric acid and elevated blood uric acid levels. This finding provides valuable information for the prevention and treatment of cardiovascular disease in patients with MAFLD. It helps to assess patients’ CVD risk more accurately, achieve more precise risk stratification, provide a basis for the development of personalized treatment and intervention plans, and adjust strategies in a timely manner to reduce mortality. Previous studies have found that high UHR values are positively associated with the risk of ischemic heart disease (26). In addition, high UHR was associated with the risk of cardiovascular mortality in diabetic and peritoneal dialysis patients (27, 28).

The increased risk of cardiovascular disease in patients with MAFLD is associated with oxidative stress, endothelial dysfunction, and systemic inflammation (17, 29). Uric acid generates reactive oxygen species (ROS) through the activation of NADPH oxidase and other pathways, leading to oxidative stress (30). In addition, uric acid inhibits nitric oxide synthase activity, resulting in reduced nitric oxide synthesis and bioavailability, leading to vascular endothelial dysfunction (31).

In contrast, HDL-C helps to mitigate oxidative damage while promoting repair and regeneration of endothelial function. Increased uric acid and decreased HDL-C result in an imbalance between pro-oxidant and anti-oxidant effects. In addition, patients with MAFLD are often associated with chronic low-grade inflammation throughout the body, which may also increase the association of UHR with cardiovascular disease. Thus, UHR may be a potential biomarker of cardiovascular disease risk in patients with MAFLD.

### Limitations

Our study still has some limitations. First, our study is based on the NHANES database, and although the weighted sample is representative of the entire U.S. population, it is still unknown whether these findings can be widely applied to other regions. Second, this study was a cross-sectional study, which could only conclude the correlation between UHR and the risk of cardiovascular disease in patients with MAFLD but could not establish a causal relationship. Finally, our study assessed patients’ CVD risk based on the Framingham Risk Score and did not specifically analyze the various CVD types and severity, and FRS was validated in general populations, not MAFLD-specific cohorts, the use of FRS in patients with MAFLD may have certain limitations. Therefore, many more refined multicentre prospective studies are still needed to confirm this finding in the future.

## Conclusion

UHR is significantly associated with 10-year CVD risk in patients with MAFLD. The higher the UHR, the higher the FRS in patients with MAFLD.UHR can be a valid biomarker for predicting 10-year CVD risk in patients with MAFLD.

## Data Availability

The original contributions presented in this study are included in this article/supplementary material, further inquiries can be directed to the corresponding author.
